# Surgery

**Published:** 1893-09

**Authors:** 


### *SELECTIONS AND ABSTRACTS.*

### SURGERY.

Edited by Dr. F. W. McRAE.

### Vomiting of Anesthesia.

Vomiting during anesthesia may be arrested by compression of the phrenic nerve and vagus. Dr. Joos, of the Cantonal Hospital at Winterthur, applies compression with the thumb on the left side, immediately above the sternal end of the clavicle. The hand is held flat on the thorax and the thumb is parallel with the clavicle. In Dr. Joos' experience the vomiting and hiccough forthwith cease. He also suggests that this treatment might be useful for the relief of seasickness.—*Medical Review.*

### Extirpation of Carcinoma of the Pylorus.

At a recent meeting of the Paris Academy of Medicine, Defontaine reported two cases in which he had successfully removed the pylorus for carcinoma. One had been operated upon a year previously and the patient had gained thirty pounds in weight. The second operation had been performed a month before. The patient was able to take nourishment on the fourth day; to arise from bed on the twelfth day, and to leave the hospital on the fifteenth day. In both cases the operation was rapidly followed by restoration to health. In a short time the yellowish, earthy appearance was replaced by a clear complexion. The results were more satisfactory than could be attained by gastro-enterostomy, as the orifice of communication between stomach and intestine remained sufficient, and the offending cause was removed.—*Medical News.*

### STRICTURE OF THE RECTUM.

I want to state, or rather reiterate, my opinion about the etiology of stricture of the rectum; that is, that the vast majority of strictures are produced by syphilis, and if I see a stricture of this portion of the gut that is not syphilitic, I strongly incline to the belief that it is cancerous. And *vice versa,* if I see a case that is suspected to be cancerous and it turns out not to be cancer, I am on record as saying, and still say, that I believe it to be syphilitic in a majority of the patients. I do not want to be understood as saying that all strictures of the gut that are not cancerous are syphilitic. I am misquoted in that particular and have tried to correct the matter in the societies, etc., but there are some who will not hear it. My position then is this, and the case in question is one that will bear me out, that if the evidence, clinical history, etc., go to prove that a stricture of the gut is not cancerous then it is generally syphilitic.—*Jos. M. Mathews, M. D.*

---

### IODOFORM IN THE TREATMENT OF TUBERCULOSIS OF BONES AND JOINTS.

Brodnitz claimed that the results of operative treatment of bone and joint tuberculosis have considerably improved since the employment of iodoform has been practiced. König explains this by assuming that tuberculosis cannot develop in quickly contracting scar tissue. Mosetig v. Moorhof attributes a specific effect to the iodoform.

For the past three years the author has used iodoform, as recommended by Bilroth, which consists of a sterilized 10 per cent. mixture with glycerin. The absolute sterilization of the mixture is of the utmost importance.

The author reports twenty cases of cold abscess subjected to this treatment. Of these nine were cured, eight had fistulæ remaining, and three were unheard from. Of twenty-five cases of bone tuberculosis operated upon, and the iodoform-glycerin mixture employed in addition, twenty were discharged cured. Nine cases of capsular tuberculosis were subjected to arthrectomy and the use of iodoform, and seven were definitely cured.

DeVos has written an exhaustive monograph on this subject. He prefers the iodoform mixed with olive oil, and carefully sterilized. It is used in exactly the same manner as iodoform-glycerin mixture.

Seventy-two cases are reported, which have been subjected to this treatment; seventy-two per cent. of these are said to be entirely cured. The duration of the treatment in the different cases was from nine to three hundred and twenty-five days. The number of injections ranged from one to twenty. The amount of iodoform used was from 2 to 65.5 grammes, and of oil, ten to three hundred and eighty-five grammes.

Improvement is shown, after beginning this treatment, by decrease of the pain and of the swelling; and new connective tissue results at the seat of the injections. This latter is said to have been observed in a patient whose knee-joint was resected after having undergone the iodoform treatment.—*Am. Jour. Med. Sciences.*

---

### The Surgical Importanceof Dust.

That atmospheric infection is, and will always remain, a factor upon which we have to reckon, Haegler, in a contribution to *Klinische Chirurgie*, proves through a very interesting series of experiments and examinations, in the rooms of the Basle Clinic; for example, in the wards before and after visits with changes of dressings, in the operating room before, during and after the clinic, in the ward for internal diseases, in the gynecological division, in the polyclinic, and finally in the rooms not specially frequented by the sick, search was made for pathological bacteria in the dust from the hair and the operating coats before and after visiting the sick, etc. It was always found that in dry and stirred air the probability of wound infection is much greater. The germs get into the dust by the drying up of wound secretions, and reach them also through the sweat, phlegm, excrement and urine. The sources cannot be completely stopped, but may be much limited.

First of all must the stirring up of dust be avoided, and airing must be done while the walls and floors are moist. In the operating room there should be as little moving around as possible, and the spray should be used before operations. The hair is to be moistened with sterile water, and the operating coat changed often, while removed dressings should immediately be thrown into water.

The importance of all these claims is well proven by experiments. The thorough dampening of the air is, as H. conclusively proves, the best way of freeing the atmosphere of germs quickly.

The so-called aseptic treatment of wounds may hereby bring practical results.—*Translated from Centralblatt für Chirurgie, by C. C. Stockard, M. D.*

---

### TREATMENT OF GONORRHEA.

The line of procedure followed out at the clinic of Blaschko, at Berlin, is thus described by Gebert (*Münchener medicin. Wochenschr.*, 1893, No. 25, p. 491). In recent cases injections are not used, but instead oil of santal is given six times a day in capsules, each containing seven and a half minims. If the patient cannot swallow capsules, the following prescription is given:

℞. Olei menth. piper., gtt. x.
Olei santal., ad. ℥ ss.
M. S.—Gtt. xv. thrice daily.

This treatment will in most cases lead to recovery in the course of three weeks. Should the secretion persist after this time injections may be begun. Of these the best are zinc sulfate, 0.5 to 100, and potassium permanganate, 1 to 200. Subsequently astringents may be employed: tannic acid, 1 to 200, or argentic nitrate, 0.03 to 100. In addition to the local treatment the diet must, of course, be properly regulated. In case of epididymitis recumbency in bed must be insisted upon and compresses applied. In cases of chronic gonorrhea of the anterior urethra injections of argentic nitrate, 1 to 2000, or stronger, are employed. In cases of chronic gonorrhea of the posterior urethra injections or applications of argentic nitrate are made.

### A Few Brief Remarks on Some of the Details Which Lend Success in Abdominal Surgery.

One secret, he said, was attention to details; another was experience; another was a good knowledge of the peritoneum, and more especially of its delicate epithelial coat. The patient should be thoroughly anæsthetized, and kept fully under during operation. The instruments should be sterilized, the surgeon's and his assistents' and nurse's hands should be carefully cleansed; the sponges should be boiled, and the abdomen around the place of incision made aseptic. It was necessary, as was done formerly, to completely dry the peritoneal cavity; the sponging was often done too vigorously, causing inflammation of the delicate membrane. Where it was indicated, Dr. Temple would flush out the cavity with plain, boiled water, moderately hot. This had not only a cleansing, but a general stimulating effect, as well as being helpful in arresting hemorrhage. He advised the drainage tube in those cases where there had been adhesions, with more or less oozing, and to be removed within the next forty-eight hours, depending on the color of the fluid. He had not had a case of hernia to follow its use. He was not in favor of giving opiates after the operation. No food should be given for twenty-four hours. To relieve thirst, a couple of ounces of water with a little salt in it was useful as an enema. The patient should be kept warm. If tympanites appeared, he would give calomel, followed by mag. sulph.—Dr. Temple in *Canadian Practitioner*.

---

### Resection of the Knee-Joint in Tubercular Disease.

Miller, of Edinburgh, gives statistics based on thirty cases of resection of the knee-joint for tubercular disease. The results were characterized as "very good" in five cases, "good" in fifteen cases, and "fair" in one case. Secondary amputations for recurrence were made in four cases. One case died shortly after operating, and in one case the disease soon returned. In the remaining cases sufficient time has not elapsed to make the results of value.

In operating, he makes an incision below the patella, deeply curved downwards toward the tuberosity of the tibia, and another straight across the center of the patella, joining the ends of the first. The upper flap is then reflected upward, the tendon of the rectus and adjacent muscular fibers divided, and the entire anterior portion of the capsule, together with the patella and an elliptical portion of skin, stripped downward and removed *en masse* by cutting through the patellar tendon and tibial attachments. The remainder of the joint is then cleaned up, bones resected, etc. It is claimed that the removal of redundant skin is in itself an advantage.

He does not believe in resecting the knee-joint of very young children, on account of the risk of obtaining an atrophic limb; amputation is preferable, as it also is when a large amount of bone must be removed. An excision should not be made when there is extensive disease of the soft parts, because recurrence is almost inevitable. Amputation is better when tubercular disease exists elsewhere; also when the patient is beyond the prime of life. Sepsis may contraindicate resection at times, but certainly not often.

The editors have recently employed Miller's method of operating in a case of extensive tubercular disease of the capsule and bones of the knee-joint, and can recommend it as an exceedingly satisfactory procedure for gaining access to and thoroughly removing the diseased tissue.—*Edinburgh Medical Journal, Univ. Medical Journal.*

---

### THE BACTERIUM COLI COMMUNE A CAUSE OF APPENDICITIS.

G. Ekehorn makes the following summary of his investigations: The primary changes in appendicitis—the catarrh and subsequent thickening of the mucous membrane and of the walls of the appendix—are the same in degree and frequency, whether fecal matter be present or not; they are not, therefore, dependent upon the latter, and we cannot with reason infer that the presence or absence of fecal matter has any causal relation with them from our present knowledge. If we admit that virulent bacteria may, after gaining entrance within the processus vermiformis, induce these primary changes and cause a catarrhal inflammation with intense swelling, edema and infiltration of the appendical wall, it is strictly in accordance with our experience of their behavior in other parts of the human organism.

In an appendix thus pathologically affected fecal matter may, through its presence, acquire grave secondary importance as touching the course of the appendicitis, partly through its pressure upon the edematous, infiltrated wall, in this way becoming a secondary cause of ulceration, gangrene and perforation, and partly through diminishing the lumen of the appendix. In consequence of such swelling of the appendical wall, a narrowing is produced at each transverse flexure of the appendix. The stenosis obtains a secondary significance, analogous to that of the fecal matter.

The pathogenic bacterium most frequently found in the colon is the most common cause of appendicitis. This is the bacterium coli commune (Escherisch). This bacterium may be pathogenic for man and become virulent to a high degree; it is pathogenic for guinea pigs and other animals used for experimental purposes.

The bacterium coli commune was present in pure culture in the contents of the processus vermiformis, in a chronic catarrhal appendicitis which was in the intermediate stage of calm, in an exacerbation of a chronic catarrhal appendicitis, and in an acute gangrenous appendicitis. It was observed, always in pure culture, in the peritoneal exudation after a perforating appendicitis, and in the pus from an intraperitoneal pelvic abscess after perforating appendicitis.

The bacteria of the colon from a chronic catarrhal appendicitis, that was, for the time being, in a state of calm, appeared to be less virulent than the bacteria from a developing or an acute appendicitis, although they were very highly virulent for guinea pigs, which is analogous to that which has been found true in regard to the bacterium coli in normal feces, on the one hand, and the alvine discharges of diarrhea and enteritis, on the other.

This, to the author, seems to prove, with the highest probability, that it has had an important rôle to act, and has not been present as a passive element.

According to the author, it may be presumed, almost to a certainty, that bacteria are the principal disturbing factors in the acute stage of appendicitis, the fecal matter or the dilatation through retarded secretions being only subordinate factors. In all probability the primary changes in appendicitis (the catarrh and the thickening of the wall) are induced by the bacteria.—*Lakareforenings forhandlingar, Universal Medical Journal.*

